# Medical Sociology

**Published:** 1909-12

**Authors:** 


**Medical Sociology.** By **James Peter Warbasse.** 12mo, pp. 355. New York and London: D. Appleton & Co. (Cloth, $2.00.)

This is a compilation of various essays, written and published by the author at different periods during the past two or three years,—now brought together in book form. From the first page to the last the work is worthy of the most careful and considerate reading. The tone is elevating, the advice wholesome. Good sense and correct reasoning characterise the entire work, which is deserving of a large place in medical literature. The world would be better if the precepts of Dr. Warbasse were more closely followed. J. A. R.

